# MultiDsk: A Ubiquitin-Specific Affinity Resin

**DOI:** 10.1371/journal.pone.0046398

**Published:** 2012-10-03

**Authors:** Marcus D. Wilson, Marco Saponaro, Mathias A. Leidl, Jesper Q. Svejstrup

**Affiliations:** Mechanisms of Transcription Laboratory, Clare Hall Laboratories, Cancer Research UK London Research Institute, Potters Bar, United Kingdom; George Washington University, United States of America

## Abstract

Ubiquitylation is a highly diverse and complex post-translational modification for the regulation of protein function and stability. Studies of ubiquitylation have, however, been hampered by its rapid reversal in cell extracts, for example through the action of de-ubiquitylating enzymes (DUBs). Here we describe a novel ubiquitin-binding protein reagent, MultiDsk, composed of an array of five UBA domains from the yeast ubiquitin-binding protein Dsk2, fused to GST. MultiDsk binds ubiquitylated substrates with unprecedented avidity, and can be used as both an affinity resin to study protein ubiquitylation, and to effectively protect ubiquitylated proteins from the action of DUBs and the proteasome in crude cell extracts. We use the resin to show that the Def1 protein becomes ubiquitylated in response to DNA damage, and to isolate ubiquitylated forms of RNA polymerase II.

## Introduction

Ubiquitin conjugation is a common post-translational modification critical in many cellular processes. Ubiquitin is a 8.5 kDa highly conserved protein that, unlike other common post-translational modifications, can be modified itself on 7 internal lysine residues, creating poly-ubiquitin chains. This can give rise of a wide variety of alternate chain topologies with many different functions. Linkages via lysine-48 (K48) are thought to be a universal signal for degradation via the proteasome [1], while lysine-63 (K63) linked chains have been implicated in many cellular processes, including DNA repair, signalling, cell-cycle control and endocytosis [2], [3]. Linkage via other lysines in ubiquitin is also possible and accounts for more than half of all poly-ubiquitin chains in *S.cerevisiae* [4]. However, no generalised function has been discovered for these alternative chain topologies. Ubiquitin is added to a substrate via a thio-ester cascade from an E1 activating enzyme to an E2 conjugating enzyme, and finally via an E3 ligase to the substrate lysine [5]. To reverse ubiquitylation and hence terminate the ubiquitylation signal, de-ubiquitylating enzymes (DUBs) can cleave specific poly-ubiquitin chains, or entirely remove ubiquitin from the target protein [6].

With so many chain topologies, the ubiquitylation signal needs to be recognised and transduced via specific effector domains. Indeed, different ubiquitin binding domains (UBDs) can recognise different chain topologies [7], [8]. These UBDs are typically small domains with relatively low affinity for ubiquitin, but they invariably co-ordinate their action with other protein-interacting motifs [9]. UBDs consist of diverse sequence and structural motifs, but share an ability to interact directly with ubiquitin. Dual ubiquitin binding domains, such as those found in the protein RAP80, can bind co-operatively to enhance the affinity for poly-ubiquitin chains [10]. Ubiquitin-associated domains (UBAs) were the first UBD-type identified [11]. They are characterised by a short region of conservation in large and otherwise unrelated sequences. The UBAs, however, share a common fold. *S. cerevisiae* Dsk2, an adaptor protein for the proteasome, contains a UBA domain at its C-terminus, and this domain binds ubiquitin with relatively high affinity [12].

Studying protein ubiquitylation *ex vivo* has been hampered by the rapid proteasomal degradation of ubiquitylated proteins, coupled with the de-conjugation of chains by DUBs, which are released from their normal constrained state upon cell lysis. In the past, chemical inhibitors of ubiquitin proteases have been used to decrease unwanted (typically non-specific) loss of ubiquitin from target proteins during preparation. However, these inhibitors are typically non-specific, short-lived in aqueous solutions, only partially inhibit ubiquitin proteases and can cause aberrant covalent modifications. For example, the inhibitor Iodoacetamide alkylates proteins, which creates an adduct equivalent to the peptide left after trypsin digesting an ubiquitylated protein, which in turn can lead to inappropriate interpretation of proteomic data [13].

For some purposes, N-terminally tagged 6×His ubiquitin combined with denaturing nickel-affinity chromatography can be used to circumvent the problem of non-specific ubiquitin chain removal by DUBs as it allows purification under denaturing conditions [14]. However, this approach is primarily appropriate for yeast as it ideally requires replacement of all ubiquitin sources in the cell with a single, tagged version, in order to avoid competition from endogenous, untagged ubiquitin [15], [16]. Isolation of native, ubiquitylated proteins has typically relied upon using UBD-containing proteins [17], [18]. Using a single UBD, however, is not very efficient, and not very specific [19]. Indeed, the affinity of ubiquitylated substrates to an individual UBD is very low (Kd ≈20–500 µM), making it an inefficient methodology [20]. Here, we describe a new high-affinity ubiquitin-binding protein, the MultiDsk. This protein reagent binds with higher affinity than any other ubiquitin-binding reagents we have tested. We show that MultiDsk can be used to enrich native ubiquitylated proteins and to protect these from the action of DUBs. The resin can also be used to efficiently purify both mono and poly-ubiquitylated proteins. Specifically, MultiDsk was used to identify the Def1 protein as a novel ubiquitylated species, modified in response to DNA damage. We also used MultiDsk to specifically isolate ubiquitylated forms of RNA polymerase II using a simple two-step protocol that can be applied to any protein of choice.

## Materials and Methods

### Generation of Expression Construct for MultiDsk Expression

**Figure 1:**
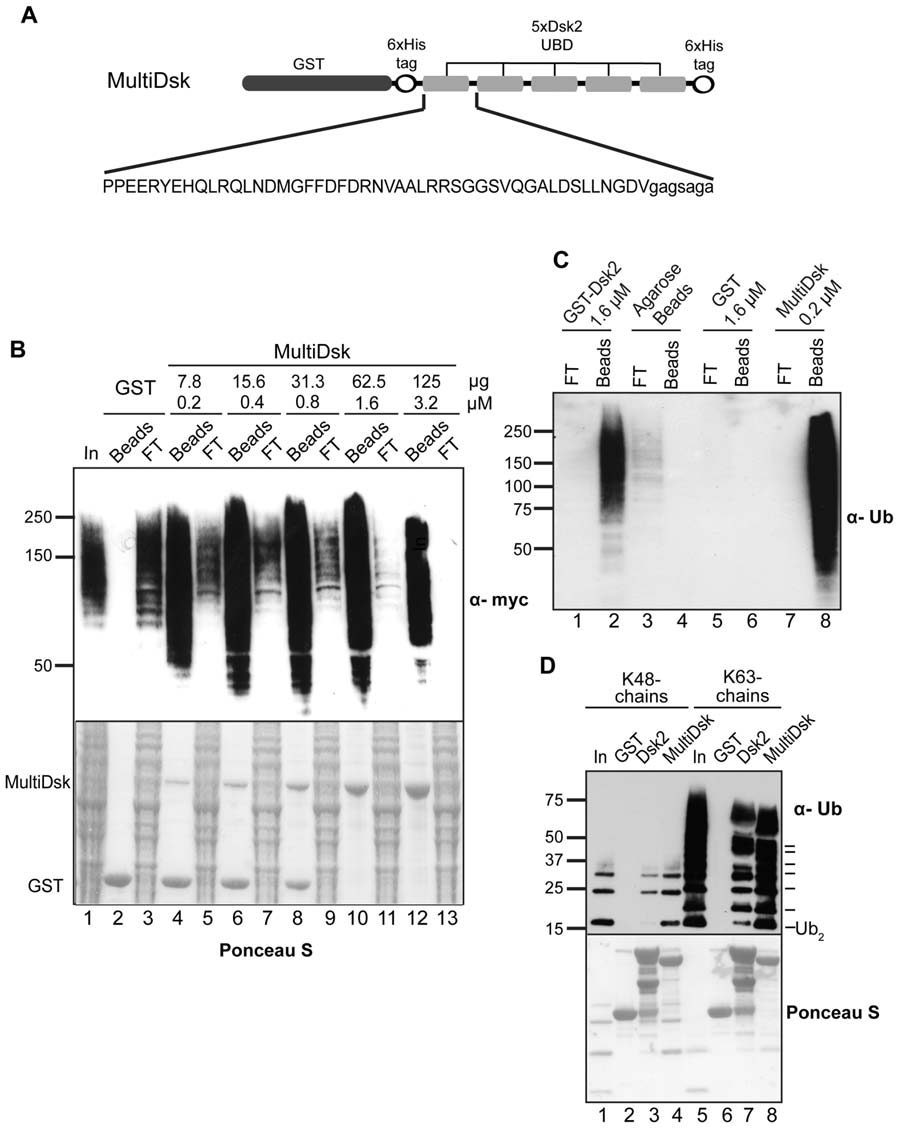
MultiDsk binds efficiently to ubiquitylated proteins. A . Schematic representation of the MultiDsk protein. **B**. 2 mg of yeast whole cell lysate from strain SUB592 expressing Myc-His-tagged ubiquitin was incubated with affinity beads. Differing amounts of GST protein, alone or as a mixture with GST-MultiDsk protein, were mixed with agarose beads so that equal amounts of total protein and bead bed volumes were used in each experiment. The flow-through not bound to the beads was retained and loaded at equivalent levels to the Input (Lane 1). After Western transfer, the membrane was stained with Ponceau S to reveal total protein in samples (lower panel), and Western blot was performed using anti-Myc antibodies to detect ubiquitylated species (upper panel). **C**. As in B, except performed on a human cell whole cell extract. 100 µg of protein was incubated with the indicated amounts of GST, GST-Dsk2, or MultiDsk protein and purified via agarose beads. **D**. As in B, but using purified ubiquitin chains. GST alone, full length GST-Dsk2 protein, or MultiDsk, bound to beads, were incubated with 100 µg synthetic K48- or K63-linked ubiquitin chains for 2 hours. After Western transfer, the membrane was stained with Ponceau S to reveal total protein in samples (lower panel), and Western blot was performed using anti-ubiquitin antibodies to detect ubiquitylated species (upper panel).

The MultiDsk construct was created from the coding sequence for the yeast Dsk2 ubiquitin binding domain (residues 327–373). This was repeated in tandem 5 times, incorporating a short 8 amino acid spacer between repeats (Figure 1A). The sequence was codon optimised and synthesised (GenScript USA, Inc), and cloned into GST fusion expression vector pGs-21a, producing pGST-MD. The DNA coding sequence and relevant protein sequence is shown in Supporting Figure 1 and Figure 1A, respectively.

### Protein Expression and Purification

MultiDsk expression plasmid pGST-MD was propagated in BL-21 (DE3) cells, selected via ampicillin. Transformed cells were grown overnight in starter culture in LB with 100 µg/ml ampicillin at 37°C. 600 ml of LB-Amp was inoculated with overnight starter culture and allowed to reach an optical density (OD_600_) of 0.6. IPTG was added to a final concentration of 1 mM, and the culture was shifted to 30°C for 4 hours. Cells were harvested by centrifugation at 6000 g for 15 min, washed once in PBS, and frozen in liquid nitrogen. Cells were lysed and protein solubilised essentially as described [21]. Briefly, thawed pellets were resuspended in STE buffer (10 mM Tris pH 8, 1 mM EDTA, 100 mM NaCl, 1× Protease Inhibitor mix [284 ng/ml leupeptin, 1.37 µg/ml pepstatin A, 170 µg/ml phenylmethylsulfonyl fluoride and 330 µg/ml benzamindine]) with N-lauryl sarcosine added to a final concentration of 1.5%. After brief sonication and centrifugation, Triton X-100 was added to the supernatant to a final concentration of 3%, to mask the sarcosine. 0.8 ml of pre-equilibrated glutathione agarose beads (GE healthcare) were added to this solution, and the slurry incubated for 2–4 hours at 4°C. The beads were washed thoroughly in STE buffer containing 500 mM NaCl and 0.1% Triton X-100, followed by a 50 mM NaCl wash in the same buffer. The protein was eluted by incubating the resin overnight with 20 mM reduced glutathione in the same buffer (4 ml elution buffer per ml resin). The purified protein was then dialysed against 50 mM HEPES pH 7.5, 150 mM NaCl, 10% glycerol.

### Yeast Cell Lysis and Ubiquitin Enrichment/protection

Exponentially growing *S. cerevisiae* strains Sub592 [16], W303-1A, or Rpb3-FLAG [22] were used throughout. Where indicated, cells were treated with UV-mimetic drug 4-Nitroquinoline 1-oxide (4-NQO) for one hour at a final concentration of 10 µg/ml in the culture medium. Cells were harvested by centrifugation at 800 g and washed once in PBS before re-suspension in D-buffer (150 mM Tris-Acetate pH 7.4, 100 mM potassium acetate, 1 mM EDTA, 0.1% Triton X-100, 10% glycerol, 1× Protease Inhibitor mix). Cells were lysed via bead-beating in MP Biosystems FastPrep-24 bioruptor and clarified by spinning at 20,000 g. Protein concentration was measured using a combination of Nanodrop and Bradford assay.

Poly-ubiquitylated substrate enrichment assays were performed using MultiDsk conjugated to GST beads (GE healthcare). Typically, 1 mg of yeast protein extract in 750 µl was incubated with 15 µl of this resin for 2 hours at 4°C before beads were extensively washed with D-buffer containing 500 mM potassium acetate, and then with the same buffer only containing 50 mM potassium acetate. Beads were resuspended in 1.5× SDS loading buffer and subjected to SDS-PAGE on BioRad Criterion 4–12%, or 3–8%, gels. Western blotting was performed as per standard procedures. Antibodies used were 4H8 (anti-Rpb1), anti-Def1 (rabbit polyclonal antibody raised against the last 350 amino acids of Def1), 9E11 (anti-myc) and P4D1 (anti-ubiquitin) (Enzo Lifesciences).

For the ubiquitylation protection assay, yeast extract was incubated with MultiDsk at 30°C for the indicated times, and then resuspended directly in SDS loading buffer prior to SDS-PAGE and Western blotting as above.

The poly-ubiquitin chain-binding assay was performed essentially as described [23]. FLAG immunoprecipitation was performed as described [22], using anti-FLAG M2 agarose (Sigma-Aldrich).

### Human Cell Lysis and Enrichment of Ubiquitylated Proteins

HEK293 cells at 70% confluency were harvested by trypsinization and washed once in PBS before being lysed in JS buffer (50 mM Hepes pH 7.5, 150 mM NaCl, 1.5 mM MgCl_2_, 5 mM EDTA, 10% Glycerol, 1% Triton X-100, 1× Protease Inhibitor mix). Cells were lysed for 15 min on ice before sonication for 10 min using a Bioruptor sonicator in a water bath, on high intensity with a 30 sec ON/30 sec OFF program, before being clarified by centrifugation for 20 min at 20,000 g. Protein concentration was measured using the Bradford assay. In order to prevent the high concentration of Triton X-100 interfering with protein binding, the extract was diluted to a final concentration of 0.25% Triton X-100. For the MultiDsk binding assay, 100 µg of total protein was incubated for 4 hours at 4°C with indicated levels of protein, followed by the addition of 25 µl bed volume of either agarose beads, or Glutathione-agarose beads (GE healthcare) for an additional 90 min at 4°C. After incubation, the beads were washed 4 times with 40 column volumes JS buffer, low Triton (50 mM HEPES pH7.5, 150 mM NaCl, 1.5 mM MgCl_2_, 5 mM EDTA, 10% Glycerol, 0.25% Triton X-100, 1× Protease Inhibitor mix), followed by SDS-PAGE on a Biorad criterion 4–12% gel. Western blotting was performed using the anti-ubiquitin antibody (P4D1, Enzo Lifesciences).

## Results

### MultiDsk Protein can be Denatured and Refolded

The engineered MultiDsk protein consists of five Dsk2 UBA domains in tandem, separated by 8-amino acid flexible linkers. An N-terminal GST tag and a C-terminal 6×His tag were also incorporated to allow detection, aid purification and allow the protein to be immobilised (Figure 1A). By obtaining a codon-optimised version of the sequence (GenScript USA, Inc), we hoped to achieve higher levels of expression, aiding purification.

Expression studies for MultiDsk were performed to maximise expression of the protein (data not shown). Under a wide range of conditions, the protein was expressed extremely well. However, it was consistently found only in inclusion bodies (Supporting Figure 2). In order to purify the protein, these inclusion bodies were first solubilised with sarcosyl, and the MultiDsk proteins were then refolded in the presence of Triton X-100 [21]. Using this approach, both the Dsk2 binding domains (as judged by their ability to bind ubiquitylated proteins) and the N-terminal GST protein (as judged by its ability to bind the glutathione resin) refolded properly. This technique allowed us to produce ∼15 mg of pure protein per litre of bacteria culture (Supporting Figure 3).

**Figure 2:**
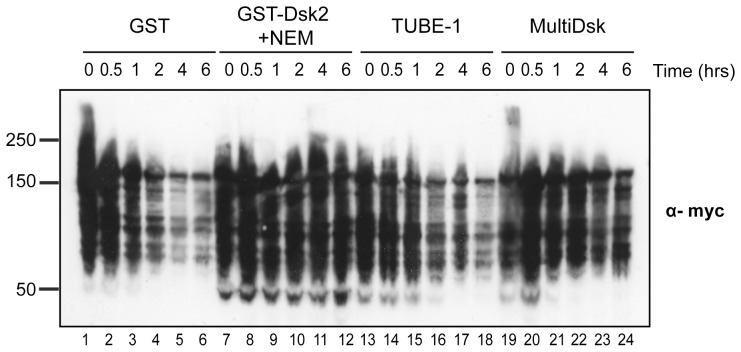
Protection of poly-ubiquitin chains in extract. Extract from strain SUB592 was incubated with equivalent amounts of GST, GST-Dsk2, commercially available TUBE-1, and MultiDsk and incubated at 30°C for the indicated time. Total protein extracts were subject to Western blot and probed using anti-myc antibody.

**Figure 3:**
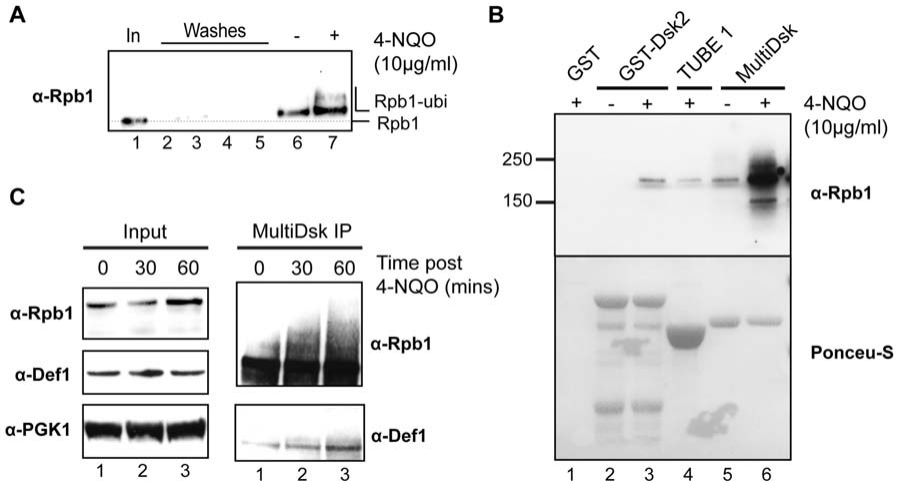
MultiDsks can be used to characterise the kinetics of ubiquitylation of a specific protein species. A. Exponentially growing yeast cells were either treated with 10 µg/ml of 4-NQO for one hour, or left untreated, as indicated. Equal amounts of extracts were incubated with MultiDsk resin. Dilute Input extract (1%) and washes from the beads were also loaded. Proteins were analysed by Western blotting using anti-Rpb1 antibody, 4H8. **B**. Exponentially growing yeast cells were either treated with 10 µg/ml of 4-NQO for one hour, or left untreated, as indicated. Equal amounts of the extracts were incubated with agarose beads loaded with GST alone, GST-Dsk2 protein, commercial TUBE1, or MultiDsk. Proteins were eluted via boiling in sample buffer and subjected to Western blot analysis using the anti-Rpb1 antibody, 4H8 (upper panel). Ponceau S staining (lower panel) shows relative amounts of affinity proteins used. **C**. Yeast cells were harvested at the indicated time after treatment with 4-NQO (10 µg/ml), incubated with MultiDsk resin, and isolated proteins were analysed by Western blotting using either 4H8, or an anti-Def1 antibody, as indicated.

### MultiDsk Protein can Bind to Ubiquitylated Proteins with High Avidity

We tested the ability of immobilised MultiDsk to purify ubiquitylated proteins from yeast extracts. In order to ease visualisation of ubiquitylated protein species in these experiments, we utilised a yeast strain where the only copy of ubiquitin is His-Myc tagged [16]. Upon isolation on glutathione beads, a strong signal for high molecular weight ubiquitin conjugates was observed in the MultiDsk pull-downs, but not in GST control (Figure 1B, compare lane 2 to lanes 4, 6, 8, 10, and 12). Remarkably, an enlarged depletion of ubiquitylated proteins occurred as the concentration of MultiDsk was increased. Only relatively modest amounts of MultiDsk was required to, more or less, completely remove all ubiquitylated proteins from the extract. At a concentration of 1.6–3.2 µM, the immobilised MultiDsk protein depleted virtually all ubiquitylated proteins from the extract (compare input (lane 1) with the flow-throughs in lane 3, lane 11, and lane 13). Not surprisingly, the resin was also effective in human cell extract, where it efficiently isolated poly-ubiquitylated protein, and was more efficient than a larger amount of the GST-Dsk2 resin we used previously [18] (Figure 1C). The binding of ubiquitylated protein by MultiDsk is also stable to washing with 500 mM NaCl, high detergent and a wide range of pHs (Supporting Figure 4). This stability allows the removal of all non-ubiquitylated proteins from their ubiquitylated counterparts. In contrast, the MultiDsk resin does not appear to enrich SUMOylated proteins (data not shown), while the possible (if unlikely) enrichment of other ubiquitin-like proteins has not been tested.

**Figure 4:**
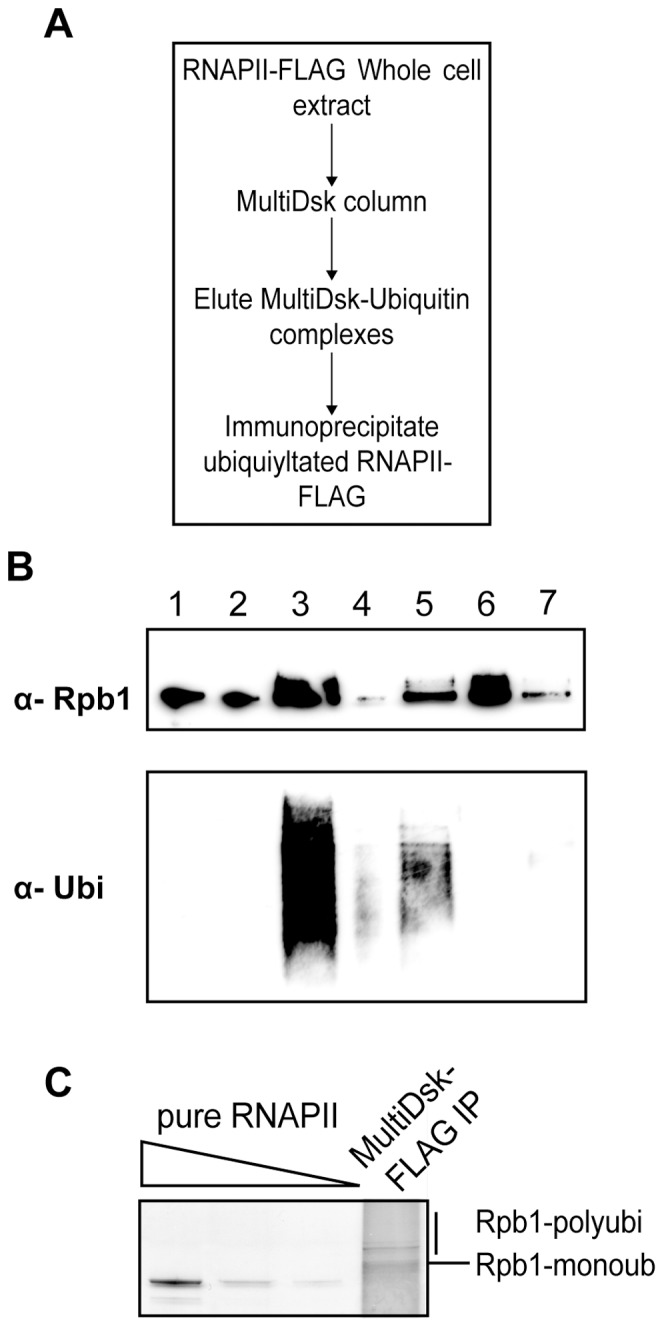
MultiDsks can be used to purify a specific ubiquitylated protein. A A schematic of purification of ubiquitylated RNAPII from 4-NQO (10 µg/ml) treated Rpb3-FLAG tagged cells. Cells were grown to logarithmic phase and treated with 4-NQO for one hour before extract was created as described earlier. **B** Western blot of two-step purification, probed with anti-Rpb1 antibody (4H8) and anti-ubiquitin antibody (P4D1). Lanes correspond to; 1- Input extract, 2- Flow through from MultiDsk resin, 3-MultiDsk resin and bound ubiquitin proteins prior to elution, 4- Gluathione beads post elution, 5- Eluted MultiDsk-ubiquitin conjugates, 6- FLAG Immunoprecipitate and 7- Flow through form the FLAG immunoprecipitate. **C** Silver stain comparing FLAG immunoprecipitate post MultiDsk enrichment (lane 6 from B) to pure Rpb3-FLAG tagged RNAPII. Different concentrations of pure RNAPII are loaded for comparison. Band at corresponding heights are labelled Rpb1 mono-ubiquitin and Rpb1 poly-ubiquitin.

Different ubiquitin-binding proteins bind different chain conformations. We investigated whether MultiDsk was capable of enriching different ubiquitylated species more or less equally. Indeed, the specificity of MultiDsk appears to be similar for purified poly-ubiquitin K48 and K63 chains (Figure 1D). Compared to the inputs, the enrichment of the isolated chains thus appears to be equivalent. The apparent discrepancy of levels between K48 and K63 chains bound is due to the ubiquitin antibody used exhibiting far higher specificity for K63 linked chains than for K48 chains, which explains the difference between the Ponceau S-detected loading and the Western blot shown (Figure 1D, lower panel). This linkage specificity has also been noted by the manufacturers (Enzo Life Sciences). MultiDsk appears to bind to all ubiquitin chain linkages. Indeed, all ubiquitylated proteins, irrespective of chain linkage, were depleted in extracts (Figure 1B, lane 13).

### MultiDsk Protein can Protect Poly-ubiquitylated Species in Extracts

We also investigated the ability of MultiDsk to protect poly-ubiquitin chains from degradation in extracts. Over a 6-hour time course at 30°C (*S. cerevisiae’s* physiological temperature), there is a gradual loss of high molecular weight ubiquitin conjugates when yeast extract is incubated with GST protein only (Figure 2, lanes 1–6). In contrast, the MultiDsk protein offered significant protection, despite being used at a relatively low concentration (1.6 µM, or 0.4 µg MultiDsk per mg crude yeast extract) with significant preservation of ubiquitylated species in solution (lanes 19–24). While MultiDsk was not as effective as using a combination of GST-Dsk2 and a high concentration of chemical inhibitor (5 mM NEM) (lanes 7–12), it was significantly more effective than the commercially available alternative TUBE-1 ([24], LifeSensors) (lanes 13–18). When ubiquitylated proteins from extract were pre-bound to the MultiDsk resin they were also protected from a wide variety of DUBs, added *in vitro* (data not shown).

### MultiDsk Protein can be Used to Identify Novel Ubiquitylated Proteins

A resin that specifically enriches ubiquitylated proteins should be useful to assess how a population of ubiquitylated proteins changes under different conditions. When only a very small sub-fraction of the total cellular pool of a particular protein is ubiquitylated, such changes cannot normally be visualised, because the Western blot signal from the unmodified version is typically much stronger. For example, this is the case for the largest subunit of RNAPII, which becomes poly-ubiquitylated in response to DNA damage ([25], [26] and references therein) in a process requiring, amongst other factors, the Def1 protein [27]. By using MultiDsk to isolate all ubiquitylated proteins, the modified protein of interest, in this case the 200 kDa Rpb1 subunit of RNAPII, can be detected by simple Western blotting. The electrophoretic mobility shift caused by the ubiquitin moiety shows that the Rpb1 signal is not due to contamination of non-ubiquitylated protein (Figure 3A, compare lane 1 with lane 6 and 7 [dotted line]). Moreover, the slower migrating poly-ubiquitylated forms of Rpb1 induced by the DNA damaging agent 4-Nitroquinoline-1-Oxide (4-NQO), can also be detected (Figure 3A, compare lanes 6 and 7).

As previously reported, ubiquitylated Rpb1 can be isolated with the (single UBA-containing) GST-Dsk2 protein [18], [26], and a similar result is obtained when using the commercially available, TUBE-1 alternative (Figure 3B, upper panel, lanes 3–4). In both cases, a single band, corresponding to mono-ubiquitylated Rpb1, is observed, but only after treatment with 4-NQO under the conditions used in this experiment and the exposure shown. In contrast, MultiDsk is much more efficient at enriching ubiquitylated Rpb1, so that not only the constitutive, mono-ubiquitylated Rpb1 form (Figure 3B, lane 5), but also the poly-ubiquitylated form induced after DNA damage can easily be observed (lane 6; see also Figure 3A, lane 7). Note that this enrichment is apparent even though a considerably smaller amount of MultiDsk protein is used compared to GST-Dsk2 or TUBE-1 (Figure 3B, lower panel; compare lanes 2–4 with 5–6).

One of the benefits of using a resin that enriches only ubiquitylated proteins is that it may assist in helping to identify novel ubiquitylation targets. In Figure 3C (upper, right panel), Rpb1 can be seen to be poly-ubiquitylated in a time-dependent manner after treatment with 4-NQO. Interestingly, we also observed the appearance of a band that cross-reacted with a Def1 antibody, this band also reproducibly increased with time upon exposure to 4-NQO (Figure 3C, lower right panel, compare lane 1 with lanes 2 and 3). This band migrates at a slightly higher position than we would expect for unmodified Def1 (not shown), supporting the idea that this is a mono-ubiquitylated form. Mono-ubiquitylated Def1 has similar kinetics of formation to that of poly-ubiquitylation of Rpb1, pointing to a possible functional connection between these modifications.

The MultiDsk resin allows the specific isolation of ubiquitylated proteins from cell extracts. Because the MultiDsk protein can easily be eluted from the glutathione resin (with free glutathione) it can also be used to purify a specific, ubiquitylated protein from extracts using a simple two-step purification protocol (Figure 4A). We used this approach to enrich ubiquitylated RNAPII from extract derived from an Rpb3-FLAG tagged strain. Total ubiquitylated proteins were enriched on MultiDsk and stringent washing allowed the removal of all non-ubiquitylated proteins. Ubiquitylated proteins were then eluted from the resin by incubation with reduced glutathione (Figure 4B, lane 5). Subsequent purification of this material via anti-FLAG M2-agarose affinity chromatography allowed the enrichment of FLAG-tagged ubiquitylated RNAPII (Figure 4B, lanes 6 and 7), and the removal of MultiDsk by high salt washing (Supporting Figure 4, lane 5). In a silver stain of the FLAG M2-immunoprecipitate side-by side with un-modified RNAPII purified by conventional means [28], bands corresponding to slower migrating forms of Rpb1 can indeed be observed (Figure 4C).

In conclusion, the MultiDsk protein binds strongly and specifically to ubiquitylated protein species and provides a useful tool for analysing protein ubiquitylation *ex vivo*.

## Discussion

The data presented here indicates that the MultiDsk resin offers an excellent alternative to previously characterised methods for studying protein ubiquitylation. MultiDsk thus not only offers the opportunity to obtain pure, native, non-tagged ubiquitylated proteins from cell extracts, but can also help ensure the maintenance of the normal ubiquitylation state via a strong protective function, inhibiting de-ubiquitylation.

By combining multiple UBDs (UBAs) from yeast Dsk2, a dramatically increased affinity for ubiquitylated proteins is achieved. The amount of ubiquitylated protein isolated with MultiDsk is thus far greater than that obtained with GST-Dsk2 [18], containing a single UBD, and it is also significantly greater than what can be isolated with commercially-available TUBE1 protein under the same conditions. In all likelihood, the MultiDsk protein minimises the entropic penalty for ubiquitin binding to multiple domains within the same molecule so that extremely efficient binding can be achieved.

Importantly, the MultiDsk resin does not appear to discriminate between different kinds of ubiquitin chains, at least not between the two most prevalent ubiquitin chains present in the cell, linked via K48 or K63. Despite previous reports indicating a preference for K48-linked chains for the Dsk2 protein [29], [30], the MultiDsk resin showed no such preference. The ubiquitin chain topology preference of the full-length Dsk2 protein may be due to the spatial orientation of its UBA domain. Indeed, the Dsk2 UBA domain alone does not show the same selectivity [31]. We deliberately designed the MultiDsk construct with a flexible linker between individual UBA domains, allowing free rotation of these domains to potentially accommodate multiple chain topologies.

MultiDsk protein can also preserve poly-ubiquitylated proteins in extracts, avoiding the need for chemical inhibition of DUBs. The protein provides a significant protective role at concentrations as low as 0.2 µM, making it suitable for use in even large-scale extract experiments, especially because large quantities of the protein can easily be produced in *E. coli*. Presumably, MultiDsk protects poly-ubiquitin chains from degradation by preventing the access of DUBs to their substrates.

Here we have shown that the resin can be used to follow the ubiquitylation status of two proteins, Rpb1 and Def1, after DNA damage. We have shown that it is possible to purify proteins ubiquitylated *in vivo* using MultiDsk resin coupled with traditional affinity-purification techniques. This allows the specific purification of the ubiquitylated protein away from its non-modified counterpart. As all steps are under native conditions the protein can be used for further functional assays.

Interestingly, Def1 has not been previously shown to be inducibly ubiquitylated using whole genome approaches, or more direct investigations [14], attesting to the high sensitivity and specificity of the MultiDsk resin. Our data suggests that Def1 is inducibly mono-ubiquitylated, with kinetics similar to that of Rpb1 poly-ubiquitylation. Since Def1 is required for RNAPII poly-ubiquitylation, it is tempting to speculate that Def1 mono-ubiquitylation plays a role in the activation of the protein. This is presently under investigation.

## Supporting Information

Figure S1 **MultiDsk DNA sequence.** DNA sequence of MultiDsk protein synthesised by Genscript. DNA was cloned into pGS-21a using BamHI/EcoRI restriction enzymes.
(TIF)

Figure S2 **MultiDsk protein is insoluble under native cell lysis conditions.** *E.coli* post-induction cell pellets were resuspended in STE buffer supplemented with the indicated chemicals. A sample was taken before the cells were lysed using extensive sonication and lysozyme treatment. The bacterial lysate was centrifuged at 20 000 g and the supernatant taken as the soluble fraction.
(TIF)

Figure S3 **Purification of MultiDsks.** MultiDsks were purified as described in materials and methods. After solubilisation and renaturation of protein not all MultiDsk is able to bind to glutathione beads. MultiDsks were eluted using reduced glutathione in solution. Gel was stained with InstantBlue (Expedeon).
(TIF)

Figure S4 **MultiDsk binding poly-ubiquitin chains is highly stable.** MultiDsk beads were incubated for 2 hours with yeast extract and spun down. They were then washed three times under the stated conditions, before elution in sample loading buffer.
(TIF)
