# Supplementary figures and images for: MultiDsk: A Ubiquitin-Specific Affinity Resin
Source: PLoS One. 2012 Oct 3;7(10):e46398. doi: 10.1371/journal.pone.0046398 (PMC3463603; doi:10.1371/journal.pone.0046398)

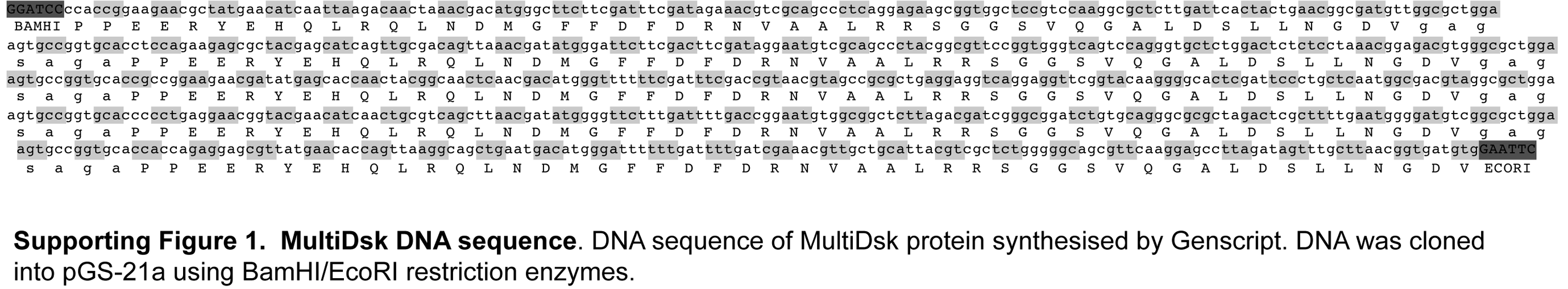

Supplement: Figure S1 — MultiDsk DNA sequence. DNA sequence of MultiDsk protein synthesised by Genscript. DNA was cloned into pGS-21a using BamHI/EcoRI restriction enzymes. (TIF) [file pone.0046398.s001.tif]

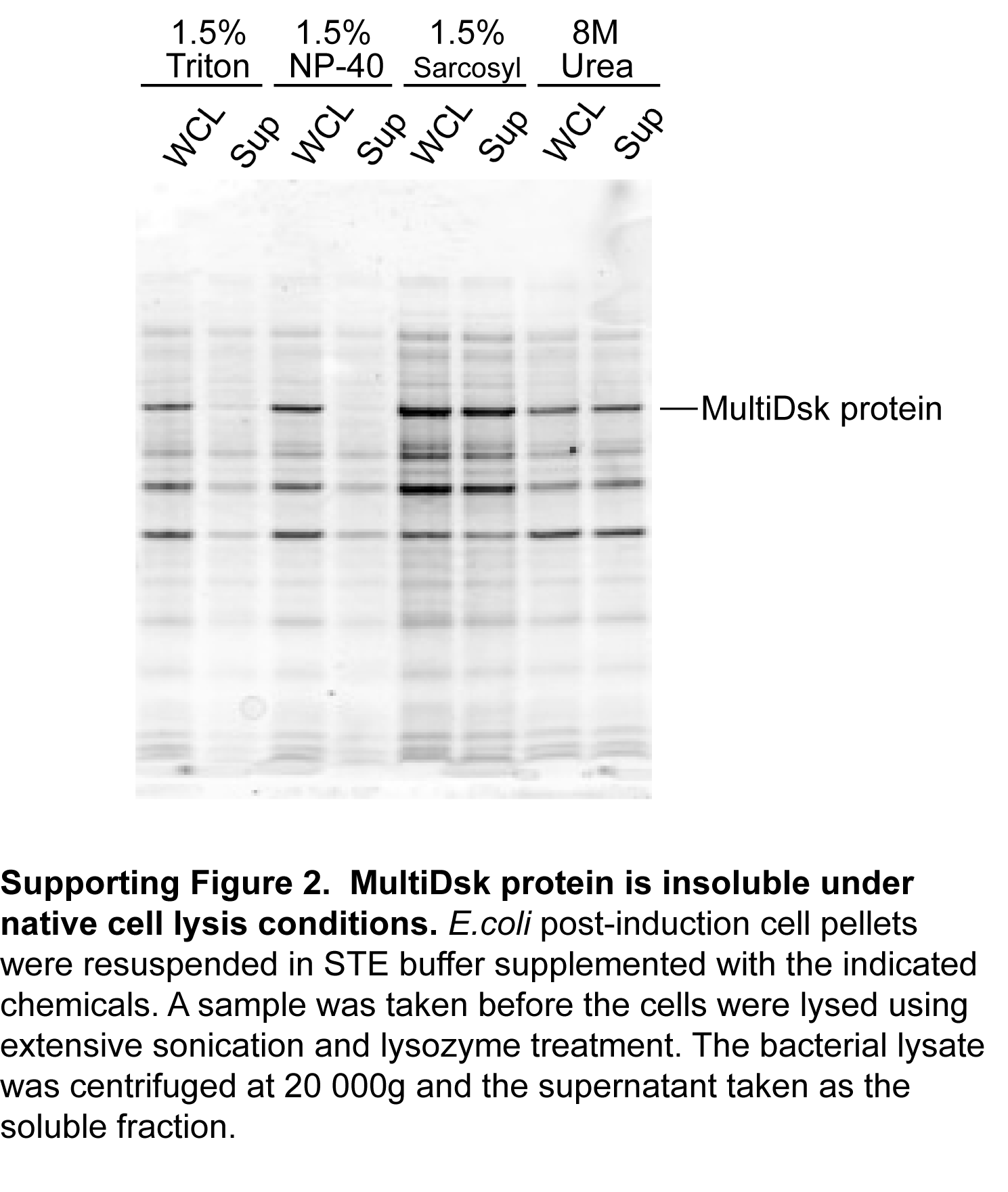

Supplement: Figure S2 — MultiDsk protein is insoluble under native cell lysis conditions. E.coli post-induction cell pellets were resuspended in STE buffer supplemented with the indicated chemicals. A sample was taken before the cells were lysed using extensive sonication and lysozyme treatment. The bacterial lysate was centrifuged at 20 000 g and the supernatant taken as the soluble fraction. (TIF) [file pone.0046398.s002.tif]

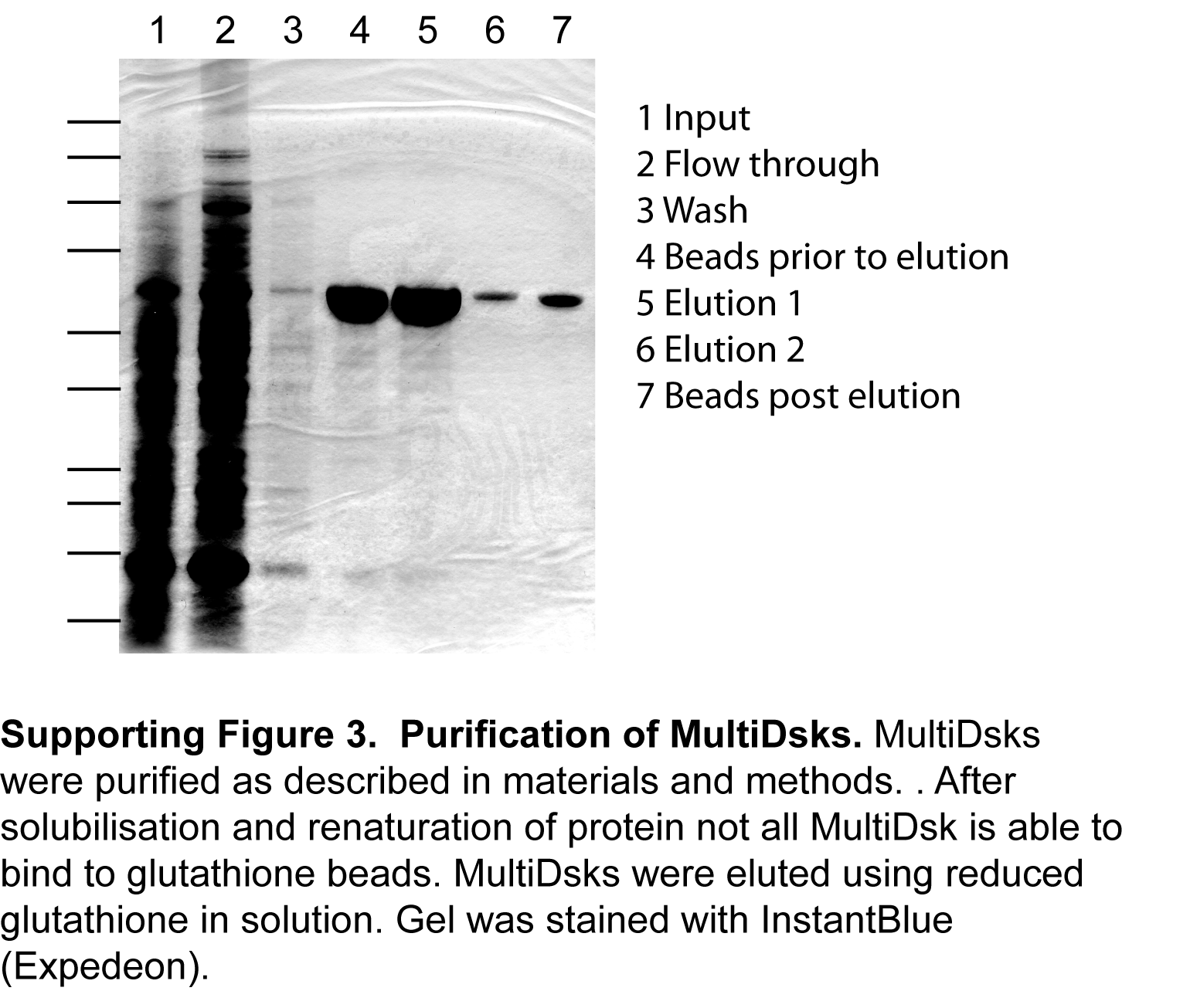

Supplement: Figure S3 — Purification of MultiDsks. MultiDsks were purified as described in materials and methods. After solubilisation and renaturation of protein not all MultiDsk is able to bind to glutathione beads. MultiDsks were eluted using reduced glutathione in solution. Gel was stained with InstantBlue (Expedeon). (TIF) [file pone.0046398.s003.tif]

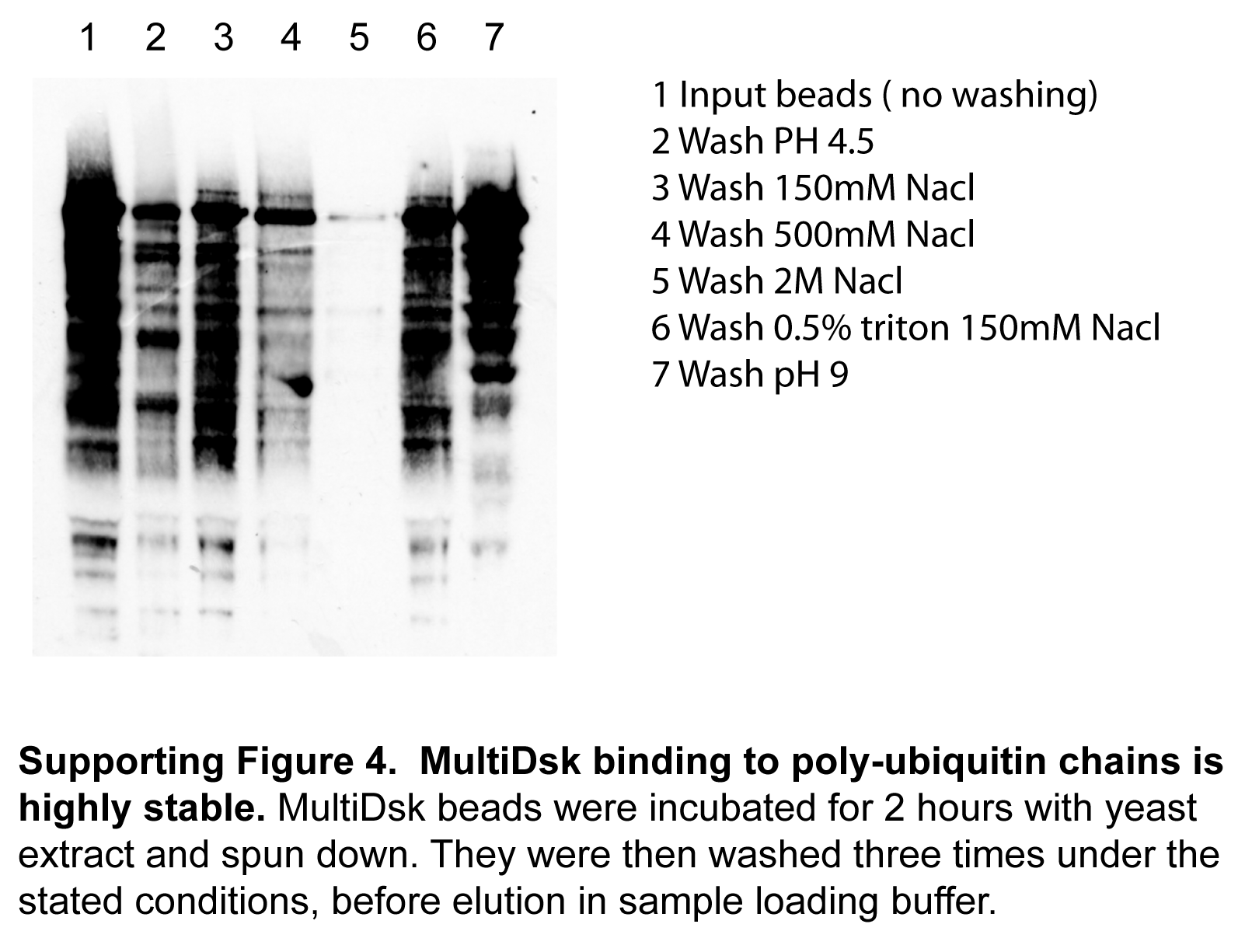

Supplement: Figure S4 — MultiDsk binding poly-ubiquitin chains is highly stable. MultiDsk beads were incubated for 2 hours with yeast extract and spun down. They were then washed three times under the stated conditions, before elution in sample loading buffer. (TIF) [file pone.0046398.s004.tif]
